# Additional Accommodation for the Insane*Superintendent F. S. White, M. D., Texas Insane Asylum; Report to the Governor for 1892.

**Published:** 1892-12

**Authors:** 


### Selections.

### ADDITIONAL ACCOMMODATION FOR THE INSANE.*

The insane population is rapidly assuming such proportions that it devolves upon the law makers to give the subject serious consideration. Were I to fail to call special attention to this matter I should feel that I had been derelict in my duty. I shall merely offer a few suggestions, which, based upon several years' experience and observation, I hope may be of some assistance in arriving at a solution of this important State problem.

The ratio of the sane to the insane, for the United States, is 1 to 545. In California, it is 1 to 216. In the District of Columbia, 1 to 189; New York, 1 to 360. If idiots be classed as insane, the ratio of the United States is raised to 1 to 269. In States where the population is very near the same as that in Texas, the ratio of insane to sane ranges from 1 to 343 to 1 to 650. It can thus readily be seen that if the population of Texas be estimated at 2,500,000, that there are within her borders at a low estimate in round numbers 4000 insane and 5000 idiots. These calculations are based largely on the census of 1880. With this array of dependent population before us, it requires no argument to establish the fact that their care and support is no small matter, but one that demands immediate attention. It is very evident to every sane man that this class must be cared for. Their support is, of course, a tax on the more favored population, and it devolves upon the State as the guardian and protector of her unfortunate citizens to provide for them in the best and most economical way possible. In devising ways and means to this end, it is expedient to take advantage of the experience of older and more advanced States in this line of charitable work. In New York, all the insane (and she has an army of them 20,000 strong) have been made wards of the State, and additional buildings are now being erected to care for them and take them all out of the miserable jails and alms-houses in which they have been so cruelly neglected for years, and give them comfortable homes, where their lives, instead of being entirely a burden, may

*Superintendent F. S. White, M. D., Texas Insane Asylum; Report to the Governor for 1892.

be rendered to a degree pleasant. Massachusetts, Pennsylvania, and in fact all the older States, are moving in the same direction. They realize the fact that in our advanced civilization this unfortunate class can not longer be neglected. Then why should Texas, the Empire State, longer refuse to make provision for all of her insane? If it is her duty to care for one of them, then it is equally her duty to care for all. It is a fundamental principle of every republican government that no discrimination shall be made between citizens. Texas has unlimited resources, but in her prosperity and greatness she must not forget and neglect her unfortunate citizens. Many who are now occupying the narrow confines of the "insane cells" of the county jails and poor-houses, reeking in filth and disease, were once honored citizens of this commonwealth. Do not the dictates of every man's conscience tell him that they deserve better treatment? I have for several years been directly in contact with this class, and it is with sorrow and regret that I hear the pitiful supplications and appeals that are made by these people for a removal from the jails and poor-houses to the asylums. In all the present asylum buildings there is accommodation for only about 1700 insane, leaving at a low estimate 2300 outside.

There must be a reform in asylum construction. The day for palatial structures for the pauper insane I hope has passed. Tne main objects in erecting asylums should be safety and comfort, and this can be attained at a much less cost than heretofore. Where is the good sense or reason in placing a class of people who have always been accustomed to the simplest dwellings, iu magnificent structures, fit for kings and queens?

Everything should be done for these people that can add anything to their comfort and that will send a ray of sunshine through their darkened and miserable lives. Give them more comfort, more amusements, better food and less palatial asylums. By these means cures may be effected in cases that would otherwise go down into oblivion and drag out a miserable living death, dead to themselves, dead to the world, and dead to their friends and loved ones.

Recent experiments in the provision for the insane have demonstrated the fact that asylums built on the cottage plan are the most economical and comfortable, and that the patients in such institutions are better and more comfortably cared for, and are better satisfied. Now for something feasible. It is evident that Texas must do something for these people. They can, if properly managed, be made very largely self-sustaining. The State should purchase in some of her agricultural districts a large tract of land and erect thereon buildings on the cottage plan sufficient to accommodate 1000 insane. Let this be for the chronic more especially, but have a few wards for the acute cases. Let the asylums now built receive the acute from throughout the State, and transfer the chronic to this new asylum, where they can be utilized on the farm and in other works. An asylum of this character can be built at a cost of from $300 to $350 per bed. With a large tract of good farming land, and buildings of this kind, the insane could be made very useful in cultivating the land, and all sorts of industries could, at a trifling cost, be established that would give all who are able employment. When constantly employed, either on farm or in shops, their health is much better, and a great pleasure is added to their lives, and their enforced confinement is rendered less irksome. The majority of them will take great interest in such work. The working classes furnish the greater number of the insane in our asylums, and it is a very valuable aid to treatment to be able to give them some good, healthful outdoor employment. It not only does the body good, but takes the mind off their delusions and troubles and directs their thoughts into different and diverting channels. I believe that it is a safe assertion that there are hundreds of asylum-made lunatics. Cases that are kept locked up in magnificent edifices, who become chronic by gazing day after day at snow white walls and polished floors. The tedious regularity of modern asylums soon becomes unbearably monotonous. This burdensome routine is detrimental to numbers of cases of insane. Change is what is required, and the best manner of securing it is by giving the inmates diversified employment and by stimulating an interest in their work. Asylums founded on this plan are a success, both economically and from a therapeutic standpoint. There are so many insane in Texas, that it behooves the State to utilize their labor as far as possible in their support. This is best for the State from a business standpoint, and is best for the insane, for it gives them outdoor exercise, and as a consequence better health and happier lives, and lessens to a very great extent the burden of their affliction.

